# Lipases and lipid droplet-associated protein expression in subcutaneous white adipose tissue of cachectic patients with cancer

**DOI:** 10.1186/s12944-017-0547-x

**Published:** 2017-08-22

**Authors:** Renata Silvério, Fábio S. Lira, Lila M. Oyama, Cláudia M. Oller do Nascimento, José P. Otoch, Paulo S. M. Alcântara, Miguel L. Batista, Marília Seelaender

**Affiliations:** 1 Cancer Metabolism Research Group, Institute of Biomedical Sciences; Department of Surgery, Faculdade de Medicina, University of São Paulo, São Paulo, Brazil; 2 Exercise and Immunometabolism Research Group, Department of Physical Education, São Paulo State University (UNESP), Presidente Prudente, SP Brazil; 3 Departamento de Fisiologia, Universidade Federal de São Paulo, UNIFESP, São Paulo, Brazil; 4 Department of Clinical Surgery, University Hospital, University of São Paulo, São Paulo, Brazil; 5 Laboratory of Adipose Tissue Biology, Center for Integrated Biotechnology, University of Mogi das Cruzes, Mogi das Cruzes, Brazil; 6 Institute of Biomedical Sciences, University of São Paulo, Av. Prof. Lineu Prestes, 1524, lab 434, São Paulo, SP CEP 05508-900 Brazil

## Abstract

**Background:**

Cancer cachexia is a multifactorial metabolic syndrome characterized by marked loss of adipose tissue and skeletal muscle. Fat loss from adipose tissue in cancer cachexia is partly the result of increased lipolysis. Despite the growing amount of studies focused on elucidating the mechanisms through which lipolysis-related proteins regulate the lipolytic process, there are scarce data concerning that profile in the adipose tissue of cancer cachectic patients. Considering its fundamental importance, it was our main purpose to characterize the expression of the lipolysis-related proteins in the white adipose tissue of cachectic cancer patients.

**Methods:**

Patients from the University Hospital were divided into three groups: control, cancer cachexia (CC), and weight-stable cancer patients (WSC). To gain greater insight into adipose tissue wasting during cancer cachexia progression, we have also analyzed an experimental model of cachexia (Walker 256 carcinosarcoma). Animals were divided into: control, intermediate cachexia (IC) and terminal cachexia (TC). Subcutaneous white adipose tissue of patients and epidydimal white adipose tissue of animals were investigated regarding molecular aspects by determining the protein content and gene expression of hormone-sensitive lipase (HSL), adipose triglyceride lipase (ATGL), comparative gene identification-58 (CGI-58), perilipin 1, leptin, adiponectin, visfatin, and tumour necrosis factor alpha (TNF-alpha).

**Results:**

We found augmented lipolysis in CC associated with increased HSL expression, as well as upregulation of ATGL expression and reduction in perilipin 1 content. In IC, there was an imbalance in the secretion of pro- and anti-inflammatory factors. The alterations at the end-stage of cachexia were even more profound, and there was a reduction in the expression of almost all proteins analyzed in the animals.

**Conclusions:**

Our findings show that cachexia induces important morphological, molecular, and humoral alterations in the white adipose tissue, which are specific to the stage of the syndrome.

## Background

Adipose tissue is the body’s largest energy reservoir and a major source of metabolic fuel. In addition to its primary role, white adipose tissue has been affirmed as a major endocrine organ, since the tissue synthesizes and secretes an array of hormones and proteins, the adipokines [1]. These adipokines allow an extensive cross talk among adipose tissue and other organs, including the brain, the liver, and the skeletal muscle.

Cancer cachexia is a multifactorial metabolic syndrome characterized by marked loss of adipose tissue and skeletal muscle, which cannot be fully reversed by conventional nutritional support and leads to progressive functional impairment [2]. While muscle wasting is associated with shorter survival time and is clearly linked with reduced quality of life, depletion of fat is also a prognostic marker for poor outcome [3].

Fat loss from adipose tissue in cancer cachexia is partly the result of increased lipolysis [4–9]. Initially, hormone-sensitive lipase (HSL) was thought to be the rate-limiting enzyme of the first lipolytic step, while we now know that adipocyte triglyceride lipase (ATGL) is the key enzyme for lipolysis initiation. HSL mRNA and protein levels are increased in the adipose tissue of patients with cancer cachexia [10–12], and studies have shown increased ATGL expression in the adipose tissue of cachectic animals [11, 13]. Moreover, ATGL-deficient mice with tumors resisted increased white adipose tissue lipolysis and maintained normal adipose and gastrocnemius muscle mass [9].

Pivotal components have also been identified at the protective interface of the lipid droplet surface and in the signaling pathways that control lipolysis. Perilipin, comparative gene identification-58 (CGI-58), and other proteins of the lipid droplet surface are currently known to be key regulators of the lipolytic machinery, protecting or exposing the TAG core of the droplet to lipases [14].

Despite growing amount of studies focused on elucidating the mechanisms through which all these lipolysis-related proteins regulate the lipolytic process, there are scarce data concerning that profile in the adipose tissue of cancer cachectic patients. Considering its fundamental importance, it was our main purpose to characterize the expression of the lipolysis-related proteins in the subcutaneous white adipose tissue of cachectic cancer patients. To gain greater insight into adipose tissue wasting during cancer cachexia progression, we have also analyzed an experimental model of cachexia. Determination of some adipokines that may be involved in cachexia progression was also performed. We describe for the first time significant alteration in the protein expression of ATGL in the subcutaneous white adipose tissue of cachectic cancer patients.

## Methods

### Experimental design

#### Patients and sample collection

Patients were recruited between November 2008 and July 2010 at University Hospital of the University of São Paulo. The inclusion criteria were as follows: 1, not having received prior anticancer treatment and 2, willingness to participate. The exclusion criteria were as follows: chemotherapy at the time of the study; continuous anti-inflammatory therapy; and kidney or liver failure, acquired immunodeficiency syndrome, inflammatory bowel disease or chronic inflammatory processes not related with cachexia, such as autoimmune disorders. Patients with body mass index (BMI) greater than 29.9 kg/m^2^ were also excluded from the study. The study was approved by the Ethics Committee from the Institute of Biomedical Sciences and by the Human Ethics Committee of the University of São Paulo Hospital (CEP-ICB/USP 1117/13, CEP-HU/USP 752/07 and 1117/13, CAAE 0031.0.198.019–07). The investigation was explained in detail to each patients, and written informed consent was obtained. They were separated into three groups, based on diagnosis after surgery. The subjects were subdivided into cancer cachexia (CC, *n* = 17), weight-stable cancer (WSC, *n* = 10) and weight-stable control (non-cancer) (control, *n* = 7). Patients were considered cachectic based on criteria from international consensus [15]. Cachexia is recognized in patients with weight loss >5% in the past 6 months or any degree of weight loss >2% in the last 6 months + BMI < 20 kg/m2. The weight stable groups were considered as those with no important weight change during the previous year and BMI < 25 kg/m2. In the cancer groups (CC and WSC) the tumour primary location was colon, stomach, pancreas and other. The control group included patients undergoing surgery for incisional hernia and chronic cholecystitis, conditions which do not present chronic inflammation.

Approximately 10 mL of blood was collected after overnight fast, within the venous access procedure for anaesthesia during the surgery. Blood was collected into tubes containing EDTA and plasma samples were obtained after centrifugation (3000 rpm for 15 min at 4 oC). Plasma was stored at −80 °C for posterior analysis. Approximately 1 g of subcutaneous white adipose tissue was collected in within a 5 min interval, similarly to that described by Agustsson et al. [8]. The adipose tissue biopsy was immediately transferred into liquid nitrogen and kept at −80 oC before analysis. This procedure presents a minimal degree of risk and does not interfere with the surgery procedure.

#### Experimental cachexia model

Male adult Wistar rats (180–200 g), obtained from the Institute of Biomedical Sciences, University of São Paulo, were maintained in a 12 h light:12 h darkness cycle, under controlled temperature conditions (22 ± 2 °C) and relative humidity at 60%, receiving water and food (NUVILAB 1, Nuvital, Brazil) ad libitum. The Ethical Committee for Animal Research from the Institute of Biomedical Sciences, University of São Paulo, approved all the adopted procedures (Protocol no. 041/2005), which were carried out in accordance with the Brazilian College for Animal Experimentation (COBEA).

Weight and food intake were assessed three times per week, always in the afternoon. To characterise different stages of cachexia progression, animals were randomly assigned into three experimental groups: control, tumour-bearing sacrificed on the seventh day (intermediate cachexia - IC) and tumour-bearing sacrificed on the fourteenth day (terminal cachexia - TC) after tumour inoculation. Walker 256 tumour cells (2 × 10^7^ cells) were injected subcutaneously into the right flank of the rats [16]. Control animals received saline injections on the same day of tumour inoculation. The experimental procedures were carried out on the seventh (IC group) and fourteenth days (control and TC groups) following tumour inoculation.

### Real time PCR

Total RNA was obtained from aliquots of 300 mg of subcutaneous or epidydimal white adipose tissue by Trizol® reagent (Invitrogen, USA) extraction according to the manufacture’s recommendations. The first strand of cDNA was generated from 2 μg of total RNA, employing a commercial kit (High Capacity cDNA Reverse Transcription Kit, Invitrogen). Polymerase chain reaction (PCR) amplification was performed in duplicates, with SYBR Green PCR Master Mix (Applied Biosystems, CA, USA) in the QuantStudio™ 12 K Flex Real Time PCR (Applied Biosystems, CA, USA), employing the primers listed in Table 1. Gene expression was normalized to 18S (humans) or GAPDH (rats). Data were calculated with the 2-ΔΔCT method and are presented as the fold change in gene expression relative to the control sample.

**Table 1:** Specific primers used for real-time PCR analysis

Gene (NCBI Genbank)	Humans	Rats
HSL (NM_005357)	Sense: AACTGCCAGCTGCCTTAAAA Antisense: TTCCCTCACGGGAGATATTG	Sense: CCTCAAAGTCAAACCCTCCA Antisense: GTGCGTAAATCCATGCTGTG
ATGL (NM_020376)	Sense: TCCTCGGCGTCTACTACGTC Antisense: CTCAATGAACTTGGCACCAG	Sense: CAACGCCACTCACATCTACG Antisense: AGCAGGCAGGGTCTTCAGTA
CGI-58 (NM_016006)	Sense: GTGCCCTAGGATTGGACAAA Antisense: GGCTCTGATCCAAACTGGAA	Sense: TGTGTCCCCTGCACTTACAA Antisense: AAAATTCAGGGCCCAAAGTC
Perilipin 1 (NM_002666)	Sense: CCCAGGAGTGACAGGAATTG Antisense: CTCGCTCCTCAAGCTTCAAC	Sense: ATCTCCTGCCACCAGACAAG Antisense: GATCCACATGGCCAGAGAGT
Leptin (NM_013076)	Sense: ACAGAAAGTCACCGGTTTGG Antisense: GTGAAGAAGATCCCGGAGGT	Sense: CCTGGTGACAATGGTCTTGA Antisense: AGCTGCAAGGTCCAAGAAGA
Adiponectin (NM_144744)	Sense: ATG ACCAGGAAACCACGACT Antisense: CACC GATGTCTCCCTTAGGA	Sense: CATCTCCTGGGTCACCCTTA Antisense: ACCCAAGGAAACTTGTGCAG
Visfatin (NM_177928)	Sense: AAAATCCAGGAAGCCAAAGA Antisense: TCCTCTGGGAATGACAAAGC	Sense:TTTCCTCGTATTTCACCTTCC Antisense: AAGCCGAGTTCAACATCCTG
18S (HQ387008)	Sense: CCTGCGGCTTAATTTGACTC Antisense: ATGCCAGAGTCTCGTTCGTT	--------------------------------------------- ---------------------------------------------
GAPDH (NM_017008)	---------------------------------------------- ----------------------------------------------	Sense: AGACAGCCGCATCTTCTTGT Antisense: CTTGCCGTGGGTAGAGTCAT

*HSL* hormone-sensitive lipase, *ATGL* adipose triglyceride lipase, *CGI-58* comparative gene identification-58, *GAPDH* glyceraldehyde 3-phosphate dehydrogenase

### Protein analysis by Western blotting

The white adipose tissue was homogenised in 1.0 mL solubilisation buffer at 4 °C (1% Triton X-100, 100 mM Tris-HCl (pH 7.4), 100 mM sodium pyrophosphate, 100 mM sodium fluoride, 10 mM EDTA, 10 mM sodium orthovanadate, 2.0 mM phenylmethylsulphonyl fluoride (PMSF), and 0.1 mg aprotinin/mL) with a Polytron (model 713 T; Fisatom Equipamentos Científicos, São Paulo, SP, Brazil). Insoluble material was removed by centrifugation for 30 min at 9000 g in a 70.Ti rotor (Beckman, Fullerton, CA, USA) at 4 °C. The protein concentration of the supernatants was measured by the BCA assay. Proteins were denatured by boiling (5 min) in Laemmli sample buffer containing 100 mM DTT, run on 8, 10, or 12% SDS-PAGE gel in a Bio-Rad miniature slab gel apparatus. The electrotransfer of proteins from gels to nitrocellulose membranes was performed for ~1.30 h/4 gels at 15 V (constant) in a Bio-Rad semidry transfer apparatus. Nonspecific protein binding to the nitrocellulose was reduced by preincubation for 2 h at 22 °C in blocking buffer (1% bovine serum albumine, 10 mM Tris, 150 mM NaCl, and 0.02% Tween 20). The nitrocellulose membranes were incubated overnight at 4 °C with antibodies against HSL, ATGL, CGI-58, perilipin 1, and alpha-tubulin (Santa Cruz Biotechnology, CA, USA) diluted 1:  1000 with blocking buffer supplemented with 1% BSA and then washed for 30 min in blocking buffer without BSA. The blots were subsequently incubated with peroxidase-conjugated secondary antibody for 1 h at 22 °C. For evaluation of protein loading, membranes were stripped and reblotted with an anti-alpha-tubulin antibody as appropriate. Specific bands were detected by chemiluminescence, and visualization/capture was performed by exposure of the membranes to RX films. Band intensities were quantified by optical densitometry of developed autoradiographs (Scion Image software-Scion Corporation, Frederick, MD, USA).

### Plasma determinations

Glucose, free fatty acids, triacilglycerol, HDL-cholesterol and total cholesterol plasma concentration was determined using commercial enzymatic kits (Labtest®, São Paulo, Brazil). LDL cholesterol was calculated according to Friedewald et al. Leptin and insulin plasma levels were measured by radioimmunoassay (Linco Research Inc. and Siemens, respectively). Adiponectin and visfatin plasma concentration was assessed by ELISA (Invitrogen,United States).

### Adipokine tissue concentration

The adipose tissue was homogenised and centrifuged at 12,000 g for 40 min at 4 °C; the supernatant was saved, and the protein concentration was determined using the BCA assay (Bio-Rad, Hercules, California) with bovine serum albumin (BSA) as a reference. Quantitative assessment of adiponectin, visfatin and TNF-alpha tissue proteins was carried out by ELISA (DuoSet ELISA, R and D Systems, Minneapolis, MN) and the concentration of leptin was carried out by radioimmunoassay (Linco Reasearch, United States). All samples were assayed as duplicates, and the mean value was reported. Results were equalised to total protein.

### Morphological analysis

Samples of the epididymal white adipose tissue were fixed in 4% paraformaldehyde, pH 7.4 for 24 h at 4oC, followed by dehydration in increasing ethanol solutions concentration (70, 95, 100%), cleared in xylene and then, embedded in paraffin. The sequential 5 μm sections obtained were stained with haematoxylin and eosin. Tissue images were obtained with a ×40 objective lens, recorded on a digital camera (DFC 295, Leica), displayed on a high resolution monitor (LG, Flatron, E1941). Morphometric aspects, area, average diameter, perimeter and shape were analysed and measured by Image Pro-Plus 6.0 (100 adipocytes per stained section).

### Statistical analysis

The statistical analysis was performed using the software SigmaStat (version 3.1, SigmaStat, SYSTAT, Point Richmond, CA). Data are expressed are as mean ± s.e.m. and analysed by one-way analysis of variance. When a significant F-value was found by one-way analysis of variance, a Bonferroni’s post hoc test was performed to demonstrate all pairwise multiple comparisons between the means. A *P* < 0.05 was considered significant.

## Results

### Clinical findings

Baseline characteristics of the patients are shown in Table 2. All the groups were matched for age and body mass index (BMI). However, the CC patients showed considerably drastic body weight loss (about 5.0-fold compared to WSC, *p* < 0.05). Free fatty acids concentration was also markedly augmented in CC when compared with control (2.6-fold, *p* < 0.05). It is notable that both cancer groups showed dyslipidemia. Total-cholesterol was higher in CC and WSC relative to control (1.5-fold and 1.6-fold, respectively, *p* < 0.05). Similarly, there was an increased concentration of LDL-C in CC (2.3-fold) and WSC (1.9-fold), both *p* < 0.05. WSC also presented higher VLDL concentration when compared with CC (21%, *p* < 0.05). Results for IL-6, IL-10, TNF-alpha, adiponectin, leptin and resistin have been published previously [17].

**Table 2:** Characteristics of study groups

	Control	CC	WSC
*n* Gender (male/female)	20 11–9	17 10–7	10 4–6
Age (years)	51.1 ± 3.6	58.4 ± 4.0	66.9 ± 4.3
BMI (kg/m^2^)	23.3 ± 0.9	20.6 ± 0.8	22.9 ± 0.7
Weight loss (%)	1.9 ± 1.0	19.9 ± 1.9^a,b^	4.0 ± 0.5
Staging, n (%)			
IA		0 (0)	3 (30.0)
IB		0 (0)	0 (0)
IIA		0 (0)	0 (0)
IIB		2 (11.7)	1 (10.0)
IIIA		4 (23.5)	1 (10.0)
IIIB		2 (11.7)	1 (10.0)
IV		9 (53.0)	4 (40.0)
CRP (mg/dL)	9.5 ± 6.8	92.3 ± 15.4^b^	90.4 ± 31.2^b^
Haemoglobin (g/dL)	12.4 ± 0.5	11.4 ± 0.4	11.3 ± 0.6
Urea (mg/dL)	36.9 ± 2.7	35.3 ± 6.5	36.9 ± 3.5
Free fatty acids (μM)	680.9 ± 30.1	1786.9 ± 551.0^b^	917.3 ± 43.3
Glucose (mM)	5.51 ± 0.2	5.3 ± 0.2	6.01 ± 0.4
Insulin (ng/mL)	0.5 ± 0.1	0.3 ± 0.1	0.6 ± 0.1
Cholesterol (mg/dL)	183.9 ± 13.7	287.3 ± 65.2^b^	300.0 ± 54.1^b^
HDL(mg/dL)	52.8 ± 3.3	42.8 ± 7.0	64.1 ± 9.9
VLDL (mg/dL)	29.5 ± 1.5	28.5 ± 0.6^a^	36.4 ± 3.0
LDL (mg/dL)	104.0 ± 11.6	239.8 ± 54.1^b^	200.3 ± 42.4^b^
Triacylglycerol (mg/dL)	147.8 ± 9.2	142.6 ± 3.8	182.1 ± 17.9

*CC* cancer cachexia, *WSC* weight-stable cancer

Values are mean ± s.d. ^a^ *P* < 0.001 versus WSC. ^b^ *P* < 0.05 versus Control

#### Adipokines gene expression

Table 3 shows mRNA levels of leptin, adiponectin and visfatin in the subcutaneous white adipose tissue of patients. Adiponectin gene expression was almost 5.0-fold higher in CC when compared with control (*p* < 0.05). In the same way, mRNA levels of visfatin were significantly higher in CC (18-fold and 5.0-fold relative to control and WSC, respectively). Leptin gene expression was higher in WSC when compared with CC (*p* < 0.05).

**Table 3:** Adipokines gene expression in the subcutaneous white adipose tissue of patients

	Control	CC	WSC
Leptin (arbitrary units)	1.00 ± 0.47	0.17 ± 0.08	2.72 ± 0.81^*b*^
Visfatin (arbitrary units)	1.00 ± 0.27	18.85 ± 1.40^a^	3.74 ± 1.34^*b*^
Adiponectin (arbitrary units)	1.00 ± 0.15	4.71 ± 1.17^a^	3.08 ± 0.78^*,*^

*CC* cancer cachexia, *WSC* weight-stable cancer

Values are mean ± s.e.m. *n* = 7–12 per group.^a^ *P* < 0.05 versus Control.^b^ *P* < 0.05 versus CC

#### Gene and protein expression of lipolysis-related proteins

**Fig. 1:**
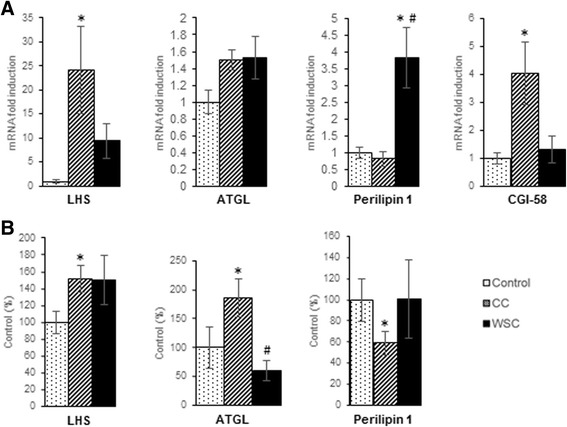
Lipolysis-related proteins expression in subcutaneous white adipose tissue from patients. *CC cancer cachexia, WSC weight-stable cancer, HSL hormone-sensitive lipase, ATGL adipose triglyceride lipase, CGI-58 comparative gene identification-58.* **a** Gene expression of HSL, ATGL, perilipin 1 and CGI-58. mRNA levels of target genes were normalized to 18 S, *n* = 7–12 per group. **b** Protein expression of HSL, ATGL and perilipin1, *n* = 8 per group. Values are mean ± s.e.m. * *P* < 0.05 versus Control, # *P* < 0.05 versus CC

The gene expression of HSL, ATGL, CGI-58 and perilipin-1 are showed in Fig. 1a. As expected, the gene expression of HSL in CC was higher than control (*p* < 0.05). On the other hand, there was no alteration in ATGL mRNA levels. However, gene expression of CGI-58, the ATGL co-activator, was increased in CC when compared with control patients (*p* < 0.05). Perilipin-1 gene expression was higher in WSC when compared with other groups (*p* < 0.05).

Similarly to HSL mRNA levels, the protein content of this enzyme was increased in CC when compared to control (*p* < 0.05) (Fig. 1b). It was also observed a higher protein content of ATGL in CC relative to control (*p* < 0.05). The perilipin-1 protein content was significantly decreased in CC when compared to control (*p* < 0.05).

### Experimental findings

#### Characterization of cachexia syndrome

Walker 256 is a well-established experimental model of cancer cachexia inducing most of the syndrome-correlated symptoms that are reported for cancer patients, as weight loss, fat depletion and decrease of food intake [18, 19]. In agreement with previous reports, body weight was decreased (after tumour tissue weight was subtracted) in tumour-bearing animals. Body weight gain was 77.2 ± 6.1 g in control animals and 20.0 ± 6.1 g in IC (*p* < 0.05 related to other groups). Nevertheless, TC presented an important weight loss (28.6 ± 12.0 g) during the experimental period (*p* < 0.05 when compared with other groups). Anorexia, an important signal of cachexia, was evident in tumour-bearing rats. Food intake was reduced in IC by 38% and in TC by 54%, when compared with control (*p* < 0.05). This reduction was significant from the fifth day after tumour inoculation and remained until the end of the experimental period (data not shown).

Cancer cachexia had significant effect on relative weight (percentage of total body weight) of epididymal and retroperitoneal white adipose tissue. Both depots were reduced in IC and TC compared with control rats. However, no differences were found regarding relative weight of mesenteric adipose tissue, nor upon liver and muscle weight. The relative weight of tumour was 3.7-fold higher in TC (3.2 ± 1.2%) when compared with IC (12.0 ± 1.9%) (*p* < 0.05).

#### Plasma measurements

As illustrated by Table 4, the adiponectin and visfatin plasma concentration was diminished in TC (*p* < 0.05 when compared with control and IC). Plasma leptin levels were decreased in IC and TC (*p* < 0.05 vs. control). Plasma free fatty acids and triacylglycerol concentrations were increased in terminal cachexia (TC) when compared with control and IC groups.

**Table 4:** Plasma measurements of experimental groups

	Control	IC	TC
*n* Adiponectin (ng/mL)	6 20.6 ± 1.4	7 19.4 ± 2.9	9 7.7 ± 0.9^a,b^
Leptin (ng/mL)	2.5 ± 0.1	1.1 ± 0.2^a^	0.7 ± 0.3 ^a^
Visfatin (ng/mL)	68.1 ± 5.8	80.2 ± 7.8	33.0 ± 6.6 ^a,b^
Insulin (ng/mL)	1.0 ± 0.0	0.6 ± 0.2	0.5 ± 0.2
Glucose (mg/dL)	100.1 ± 4.2	96.7 ± 5.3	105.1 ± 6.3
Free fatty acids (μM)	629.6 ± 94.5	758.9 ± 37.0	1423.8 ± 66.5 ^a,b^
Triacylglycerol (mg/dL)	64.9 ± 8.4	124.1 ± 26.4	236.4 ± 36.5 ^a,b^

*IC* intermediate cachexia, *TC* terminal cachexia

Values are mean ± s.e.m.^a^ *P* < 0.05 versus Control.^b^ *P* < 0.05 versus IC

#### Morphological aspects and morphometrical analysis

**Fig. 2:**
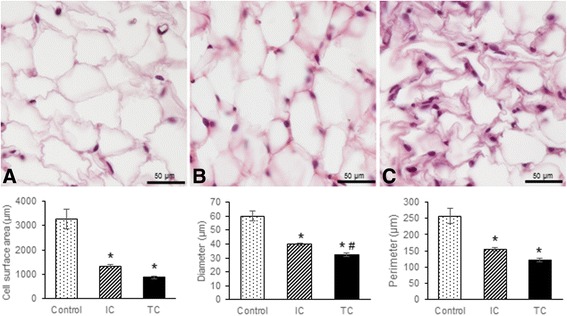
Morphological characteristics and morphometric analysis of epidydimal white adipose tissue during cachexia progression. *IC intermediate cachexia, TC terminal cachexia.* Morphometric analysis of cell surface area, diameter and perimeter of adipocytes from different experimental groups. (**a**) Control, (**b**) intermediate cachexia, (**c**) terminal cachexia. Note adipocytes of various sizes from IC and shrunken and shapeless adipocytes in TC. Values are mean ± s.e.m. * *P* < 0.05 versus control; # *P* < 0.05 versus IC

We examined whether the dramatic reduction in adipose tissue was apparent at the microscopic level. Haematoxylin/Eosin staining revealed important alterations between the epididymal white adipose tissue of control and cachectic animals. Adipocyte size was dramatically reduced in IC and TC when compared with control (*p* < 0.05) (Fig. 2). Moreover, tissue analysis showed cell size variation in IC (Fig. 2b) and shrunken and polygonal adipocytes in TC (Fig. 2c). Marked changes in the extracellular matrix were also observed in terminal cachexia, suggesting impairment of cellular function.

#### Gene and protein expression of adipokines

**Fig. 3:**
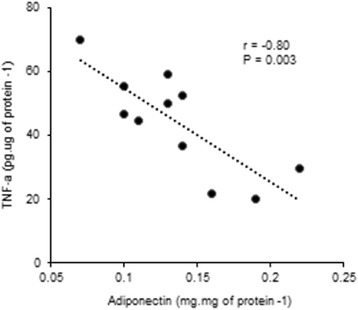
Correlation between TNF-alpha and adiponectin levels in the epidydimal white adipose tissue from animals. *n* = 8–12 per group. Values are mean ± s.e.m.

The reduction in the epididymal white adipose tissue weight was accompanied by drastic changes in gene and protein expression of adipokines (Table 5). In the middle-stage of cachexia (IC) there was a reduction in leptin gene expression and an increase in adiponectin and visfatin mRNA levels (Table 5). At the end-stage of the syndrome (TC) there was a great reduction of adiponectin mRNA, and visfatin gene expression returned to control values, while leptin showed even lower mRNA levels (Table 5). In addition, we also evaluate the protein expression of leptin, adiponectin, visfatin and TNF-alpha (Table 5). There was a decrease in leptin and adiponectin tissue content in IC (*P* < 0.05 vs. control). At the end-stage of cachexia (TC) the response was different: the leptin tissue concentration was maintained and adiponectin showed an even more pronounced decrease (*P* < 0.001 vs. control and IC). The TNF-alpha tissue concentration was 2.0-fold higher in IC (*P* < 0.05 vs. control) and 2.5-fold higher in TC (*P* < 0.05 vs. control and IC). Moreover, the adiponectin concentration was inversely correlated with the TNF-alpha level in the epididymal white adipose tissue (*r* = − 0.80, *P* = 0.003) (Fig. 3).

**Table 5:** Adipokines gene and protein expression in the epidydimal white adipose tissue from animals

	Control	IC	TC
Gene expression			
Leptin (arbitrary units)	1.00 ± 0.08	0.57 ± 0.11^*a*^	0.15 ± 0.08^a,b^
Visfatin (arbitrary units)	1.00 ± 0.18	5.1 ± 1.63^*a*^	1.17 ± 0.32^b^
Adiponectin (arbitrary units)	1.00 ± 0.14	3.68 ± 0.26^*a*^	0.18 ± 0.09^,a,b^
Protein expression			
Leptin (ng.mg of protein^−1^)	3.57 ± 0.53	1.91 ± 0.34^*a*^	1.50 ± 0.16^a^
Visfatin (ng.mg of protein^−1^)	27.55 ± 2.59	31.66 ± 2.83	12.67 ± 5.54^,a,b^
Adiponectin (mg.mg of protein^−1^)	0.21 ± 0.01	0.14 ± 0.01^*a*^	0.09 ± 0.01^a,b^
TNF-alpha (pg.μg of protein^−1^)	23.86 ± 2.87	44.52 ± 2.88^*a*^	57.61 ± 3.33^a,b^

*IC* intermediate cachexia, *TC* terminal cachexia

Values are mean ± s.e.m. *n* = 8–12 per group. ^a^ *P* < 0.05 versus Control. ^b^ *P* < 0.05 versus IC

#### Gene and protein expression of proteins involved in lipolysis

**Fig. 4:**
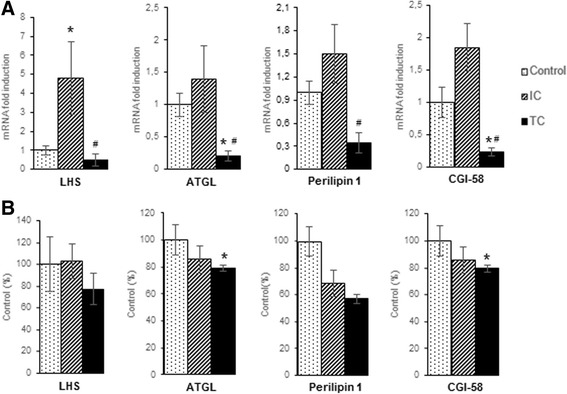
Lipolysis-related proteins expression in epidydimal white adipose tissue during cancer cachexia progression. *HSL hormone-sensitive lipase, ATGL adipose triglyceride lipase, CGI-58 comparative gene identification-58, IC intermediate cachexia, TC terminal cachexia.* **a** Gene expression of HSL, ATGL, perilipin 1 and CGI-58. mRNA levels of target genes were normalized to GAPDH, *n* = 6–12 per group. **b** Protein expression of HSL, ATGL, perilipin 1 and CGI-58, *n* = 6–9 per group. Values are mean ± s.e.m. * *P* < 0.05 versus Control, # *P* < 0.05 versus IC

To examine the contribution of lipases to mobilization of triacylglycerol, the gene and protein expression of HSL, ATGL, CGI-58 and perilipin 1 was carried out in the epididymal white adipose tissue (Fig. 4). The HSL gene expression was higher in IC, while other proteins were unaffected. At the end-stage of cachexia (TC), the HSL gene expression was similar to control values and the other proteins mRNA levels were reduced. However, protein expression showed a different pattern: ATGL and CGI-58 were reduced, while perilipin 1 presented a tendency for reduction (*P* = 0.06 vs. control) in TC.

## Discussion

Clinical studies documenting enhanced lipolysis in cachectic cancer patients implicate lipases such as ATGL and HSL in the development of cachexia [9]. Our results show, for the first time, profound changes in ATGL and perilipin-1 protein expression in the subcutaneous white adipose tissue of cachectic cancer patients. Moreover, the CGI-58 modulation during cancer cachexia has never been shown before.

Lipolysis is tightly regulated through ATGL and HSL, of which ATGL is the initial rate-limiting enzyme in the conversion of triacylglycerol into glycerol and free fatty acids [20]. In agreement with previous reports, we observed a great increase in HSL gene and protein expression in the subcutaneous white adipose tissue in cancer cachexia. However, studies have shown that while HSL makes some contribution to enhanced lipolysis in cancer cachexia, ATGL-mediated lipolysis of triglycerides appears to predominate [9, 13]. The absence of ATGL and, to a lesser degree, HSL reduces fatty acids mobilization, retains white adipose tissue and muscle mass, and prevents cancer cachexia [9]. Consistent with these data, we showed for the first time an increased ATGL protein content in the subcutaneous white adipose tissue of cancer cachectic patients. ATGL activity is regulated through interaction with its co-regulator CGI-58, but in the basal state CGI-58 is sequestered in a complex with perilipin 1 [20]. Downregulation of perilipin due to chronic TNF-alpha treatment correlates with stimulation of lipolysis [2, 21]. Thus, the higher CGI-58 mRNA level concomitant with the reduced perilipin 1 content in the subcutaneous white adipose tissue of cachectic patients strongly confirm the crucial role of ATGL in the lipolytic process in such condition.

Studies of lipid metabolism in cachexia have generally focused on later stages of the disorder when severe loss of adipose tissue has already occurred. Therefore, we also studied adipose tissue in the Walker-256 carcinosarcoma model to identify the contribution of these lipolysis-related proteins to the fat mass loss during cachexia progression. In contrast to the aforementioned results, in cachectic cancer animals we detected an increase in the mRNA level of HSL only during the middle of the experimental period (IC), without any variation in HSL protein content. Interestingly, the ATGL mRNA and protein levels were markedly reduced at the end-stage of the syndrome (TC), as well as CGI-58 expression.

Recently our study group demonstrated an important structural modification in the subcutaneous white adipose tissue of cachectic patients, including remodeling of the extra-cellular matrix, increased collagen amounts, adipocyte size reduction, and adipose tissue atrophy [22]. Some aspects of this structural modification of the white adipose tissue were also observed in our experimental model. Evidence indicates that adipose tissue remodeling in obesity is closely associated with adipose tissue function [23]. Changes in the number and size of the adipocytes affect the microenvironment of expanded fat tissues, accompanied by alterations in adipocyte death, local hypoxia, and adipokine secretion [24].

Adipokines play a role in a wide variety of physiological or pathological processes, including immunity and inflammation, in addition to having significant effects on metabolism. The association between cancer cachexia and adipokine level alterations has been reported [17, 25, 26]. Increased production of lipolytic factors by the adipose tissue, such as TNF-alpha, contributes to the disrupted lipid metabolism and increased lipolysis in cancer cachexia [2, 8]. Thereby, the white adipose tissue is both a victim and a sponsor of cachexia-related systemic inflammation [7, 27].

Leptin has a pivotal role in the control of food intake, and there are controversial results on leptin and cancer cachexia in literature [19, 26, 28, 29]. We found a reduced leptin plasma level as well as mRNA and protein content in the epididymal white adipose tissue of cachectic animals. Previously, our study group also showed a decrease in leptin plasma level and mRNA content in the subcutaneous white adipose tissue of cancer cachetic patients [17]. It has been shown that pro-inflammatory cytokines, such as IL-1beta and TNF-alpha, decrease leptin gene and protein expression in adipocytes from subcutaneous white adipose tissue of humans [30]. This interaction might influence the circulating leptin levels and thereby the adipose tissue to brain signaling.

Insulin resistance is an early event in cachexia development and may be a role in cachexia pathogenesis [11]. According to literature, high plasma levels of leptin are related to insulin resistance, and leptin reduces insulin sensitivity in isolated adipocytes [31]. Therefore, it seems paradoxical that a positive association between low serum leptin levels and insulin resistance was found in cancer patients [32].

A recent study showed that insulin treatment prevented the increased expression of ATGL and HSL in the retroperitoneal white adipose tissue, as well as increased the food intake of tumor-bearing rats [11]. Visfatin (nicotinamide phosphoribosyltransferase – NAMPT) was described as an insulin-mimetic adipokine secreted from visceral fat [33]. We demonstrated a marked increase on visfatin gene expression in white adipose tissue in experimental intermediate cachexia (IC), and the same was observed in the subcutaneous white adipose tissue of cancer cachexia patients. Visfatin can improve insulin sensitivity [34], and we therefore hypothesize that this increase may be an attempt to compensate the insulin insensitivity observed in cachexia syndrome. However, its plasma and protein levels in white adipose tissue were not upregulated in these animals and patients. Moreover, animals at the end-stage of the syndrome (TC) showed reduced visfatin plasma levels as well as mRNA and protein content in white adipose tissue.

Adiponectin is another adipose-specific protein that is important to regulate insulin resistance and has a potent anti-inflammatory function. It circulates in high concentration in the plasma, and its levels are reported to be inversely correlated with body weight. Nevertheless, the role of this adipokine in the setting of cancer cachexia is controversial. We showed higher adiponectin plasma levels in cachectic cancer patients [17], as well as other study groups [26, 35]. However, inconsistent results show low adiponectin levels [25, 36], or even absence of correlation in cachexia patients [37]. The higher mRNA content in subcutaneous white adipose tissue of CC is consistent with our previous report [17]. Opposite results were found in experimental cancer cachexia. Using the experimental model, we showed that adiponectin levels increased in the fourth day after tumor inoculation and, subsequently, that level decreased (similar to cachectic cancer patients). This increase may represent a compensatory response aimed at controlling the syndrome progression.

The pro-inflammatory cytokine TNF-alpha, which is synthesized in both adipocytes and infiltrated macrophages [38], regulates the function and development of white adipose tissue by stimulating lipolysis and inhibiting lipogenesis and adipogenesis [39]. Furthermore, TNF-alpha has regulatory functions on adiponectin in the human visceral white adipose tissue, and TNF-alpha levels are elevated in states of insulin resistance [40]. We verified a progressive increase on TNF-alpha protein content in the visceral white adipose tissue of cachectic rats. Besides, there was a negative correlation between adiponectin and TNF-alpha levels in the visceral white adipose tissue of these animals. These data indicate a modulation of the chronically and locally produced TNF-alpha upon adiponectin synthesis by the white adipose tissue of cachectic animals.

## Conclusions

Despite potential species-specific (human vs. rat) and depot-specific (subcutaneous vs. visceral) response, it is possible to infer that the data from cancer cachectic patients are similar to intermediate cachexia. Moreover, we hypothesize that the alterations in terminal cachexia animals are consistent to the alterations observed in patients with refractory cachexia.

**Fig. 5:**
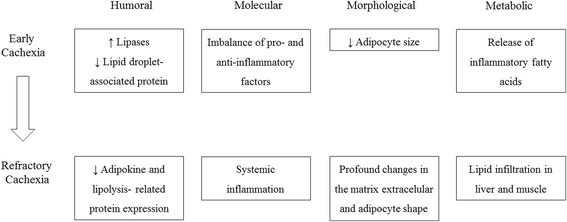
Changes along cachexia progression

The main findings of this study are integrating in Fig. 5. In summary, we found augmented lipolysis in cachectic patients associated with increased ATGL and HSL protein expression, as well as reduction in perilipin 1. On the intermediate stage of the syndrome, similar to the clinical study, there was an imbalance in the secretion of pro- and anti-inflammatory factors. At this stage, it seems that the modulation of adipokines gene expression is an attempt to unsuccessfully preserve fat mass. The alterations at the end-stage of cachexia were even more profound, and the marked decrease in adipokine and lipolysis-related protein expression, associated with changes in the extracellular matrix and adipocyte shape, suggests impairment of cellular function in terminal cachexia. Our findings show that cachexia induces important morphological, molecular and humoral alterations in the white adipose tissue, which are specific to the stage of the syndrome.

## Abbreviations

ATGL: Adipose triglyceride lipase
BMI: Body mass index
CC: Cancer cachexia patients
CGI-58: Comparative gene identification-58
CRP: C-reactive protein
GAPDH: Glyceraldehyde 3-phosphate dehydrogenase
HDL: High-density lipoprotein
HSL: Hormone-sensitive lipase
IC: Intermediate cachexia
LDL: Low-density lipoprotein
TC: Terminal cachexia
VLDL: Very low-density lipoprotein
WAT: White adipose tissue
WSC: Weight-stable cancer patients
